# Work of Breathing for Aviators: A Missing Link in Human Performance

**DOI:** 10.3390/life14111388

**Published:** 2024-10-28

**Authors:** Victoria Ribeiro Rodrigues, Rheagan A. Pratt, Chad L. Stephens, David J. Alexander, Nicholas J. Napoli

**Affiliations:** 1 Human Informatics and Predictive Performance Optimization (HIPPO) Lab, Department of Electrical and Computer Engineering, University of Florida, Gainesville, FL 32608, USA; victoria.ribeiro@ufl.edu (V.R.R.); rheagan12alexa@gmail.com (R.A.P.); 2 Breathing Research and Therapeutics Center, College of Public Health and Health Professions, University of Florida, Gainesville, FL 32603, USA; 3 United States Air Force, Washington, DC 20330-1126, USA; 4 Langley Research Center, National Aeronautics and Space Administration (NASA), Hampton, VA 23666, USA; chad.l.stephens@nasa.gov; 5 Johnson Space Center, National Aeronautics and Space Administration (NASA), Houston, TX 77058, USA; david.j.alexander@nasa.gov

**Keywords:** work of breathing (WoB), high inspiratory frequencies, anti-G straining maneuver, aviation, non-sinusoidal breathing

## Abstract

In this study, we explore the work of breathing (WoB) experienced by aviators during the Anti-G Straining Maneuver (AGSM) to improve pilot safety and performance. Traditional airflow models of WoB fail to adequately distinguish between breathing rate and inspiratory frequency, leading to potentially inaccurate assessments. This mismatch can have serious implications, particularly in critical flight situations where understanding the true respiratory workload is essential for maintaining performance. To address these limitations, we used a non-sinusoidal model that captures the complexities of WoB under high inspiratory frequencies and varying dead space conditions. Our findings indicate that the classical airflow model tends to underestimate WoB, particularly at elevated inspiratory frequencies ranging from 0.5 to 2 Hz, where resistive forces play a significant role and elastic forces become negligible. Additionally, we show that an increase in dead space, coupled with high-frequency breathing, elevates WoB, heightening the risk of dyspnea among pilots. Interestingly, our analysis reveals that higher breathing rates lead to a decrease in total WoB, an unexpected finding suggesting that refining breathing patterns could help pilots optimize their energy expenditure. This research highlights the importance of examining the relationship between alveolar ventilation, breathing rate, and inspiratory frequency in greater depth within realistic flight scenarios. These insights indicate the need for targeted training programs and adaptive life-support systems to better equip pilots for managing respiratory challenges in high-stress situations. Ultimately, our research lays the groundwork for enhancing respiratory support for aviators, contributing to safer and more efficient flight operations.

## 1. Introduction

### 1.1. Motivation

Aeronautic environments present unique challenges for the human cardio-respiratory system due to factors such as (1) high altitude flight (typically above 10,000 feet), (2) aircraft maneuvers that subject pilots to varying gravitational forces and chest constriction [1,2], and (3) alterations in air temperature and humidity. These special challenges can greatly impact cognitive workload and in-flight performance [3,4]. Aviators that are particularly at risk of experiencing these factors are jet and aerobatic pilots. Their high-performance aircraft fly at intense speeds and are capable of performing rapid directional changes. Although these aviation platforms are modern-day engineering feats, they present unique physiologic challenges to their operators. One must consider these challenges when studying human factors of breathing that may impair ventilatory homeostasis [5,6]. Monitoring these respiratory challenges in real-time is crucial, but it is nearly impossible to measure such physiological demands directly within the aircraft. This is due to equipment limitations and the invasive nature of some of the sensors, such as esophageal pressure. Only recently has nascent technology been developed to noninvasively measure respiratory metrics, such as pressure and airflow, without the need for cumbersome or intrusive devices, enabling new insights into how these environments impact breathing dynamics within aviation [6,7]. This research presents noninvasive respiratory airflow models that estimate the work of breathing, which can potentially be utilized to analyze respiratory flight data and understand physiological limitations.

To combat the many physiological limitations pilots face in extreme aviation environments and to optimize their performance, supplemental techniques, physiological monitoring, and specialized equipment have been developed [6,8,9,10,11,12]. For example, the flight oxygen mask supplies pilots with supplemental oxygen and allows them to communicate with supportive air crew [13]. This supplemental oxygen from the flight oxygen mask lowers the risk of hypobaric hypoxia, wherein reduced oxygen is delivered to the pilot’s tissues (e.g., the brain) due to the decreased partial pressure of O_2_ in the atmosphere at high altitude. During high G maneuvers, pilots are also at risk of stagnant hypoxia due to blood pooling in the lower limbs. These conditions can negatively impact cognitive function, induce stress and anxiety, and, if not addressed promptly, may lead to loss of consciousness [14,15,16]. It is important to note that there are additional physiological limitations that aviators must combat that will not be mentioned here. For this paper, we focused on reviewing physiological limitations pertaining to breathing only. For a full review of additional limitations, please see [17].

An additional strategy to combat physiological limitations in extreme aerospace environments taught to aviators is the anti-G straining maneuver (AGSM). The AGSM increases pilots’ +G_z_ tolerance and reduces the risk of gravitational loss of consciousness (GLOC). This is due to an increase in gravitational forces preventing the brain from receiving adequate oxygen-rich blood flow, causing GLOC. As a countermeasure to GLOC, AGSM increases aortic blood pressure by two components. One component is the contraction of the leg and abdominal muscles to increase blood pressure and blood flow to the heart, force blood flow to the brain, and prevent blood from pooling in the lower extremities. The other component is a modified Valsalva maneuver to increase intra-thoracic pressure, facilitating venous return and maintaining cranial blood pressure [7,18]. Before the +G_z_ onset, a preparatory breath is taken, filling a little more than half of the lung’s capacity without significant chest or shoulder movement. A forced expiratory breath against the closed glottis should be maintained for 2.5 to 3.5 s to increase intra-thoracic pressure. Then, a rapid exhale and inhale are performed to promote gas exchange, lasting from 0.5 to 1 s. This cycle of forced expiratory breath and rapid inhale and exhale should continue every 2.5 to 3.5 s during +G_z_ exposure [7,18,19]. The isometric contractions combined with the breathing techniques within the AGSM must be applied to maintain an aviator’s consciousness [20]. This maneuver, however, drastically deviates from the normal breathing pattern of a human and imposes challenges that are often neglected by the community.

This paper aims to investigate the impact of the high-velocity respiratory flow (e.g., AGSM breathing) and the imposed additional dead space (e.g., pilot mask) in the respiratory work performed by pilots. Understanding the physiological effects of the AGSM is a critical element that can be used to design breathing systems to facilitate counteracting the effects of +G_z_ flight. The presentation of more accurate respiratory models will raise awareness of the actual high respiratory work being imposed during AGSMs, which can lead to discussions on how to decrease the risk of dyspnea and improve cognitive task performance in the cockpit. The AGSM is still an essential element of pilot protection for GLOC. The specific requirements for and the effects of breathing systems on the performance of the AGSM are critical elements to designing compatible breathing systems. Research regarding respiratory work during AGSMs can lay the foundation for creating such standards.

### 1.2. Prior Work

Work of Breathing (WoB) is the energy expended by the respiratory muscles to maintain appropriate alveolar ventilation [21] and is usually measured as the area under the curve of the pressure–volume loop. This measurement assesses the level of effort under specified conditions and is useful for the trajectory of performance, especially in complex flying environments. In order to accurately measure WoB, however, the esophageal pressure must be measured, which requires the placement of a pressure catheter or balloon within the esophagus. This invasive measurement is not feasible to implement in the aeronautic environment, where only airflow and mouth pressure can be collected. Thus, any WoB calculation made using the mouth pressure may not reflect the true total work being performed but rather only account for the external work and neglect the rest [7,22]. Work of Breathing (WoB) is the energy expended by the respiratory muscles to maintain appropriate alveolar ventilation [21] and is usually measured as the area under the curve of the pressure–volume loop. This measurement assesses the level of effort under specified conditions and is useful for the trajectory of performance, especially in complex flying environments. In order to accurately measure WoB, however, the esophageal pressure must be measured, which requires the placement of a pressure catheter or balloon within the esophagus. This invasive measurement is not feasible to implement in the aeronautic environment, where only airflow and mouth pressure can be collected. Thus, any WoB calculation made using the mouth pressure may not reflect the true total work being performed but rather only account for the external work and neglect the rest [7,22].

**Figure 1:**
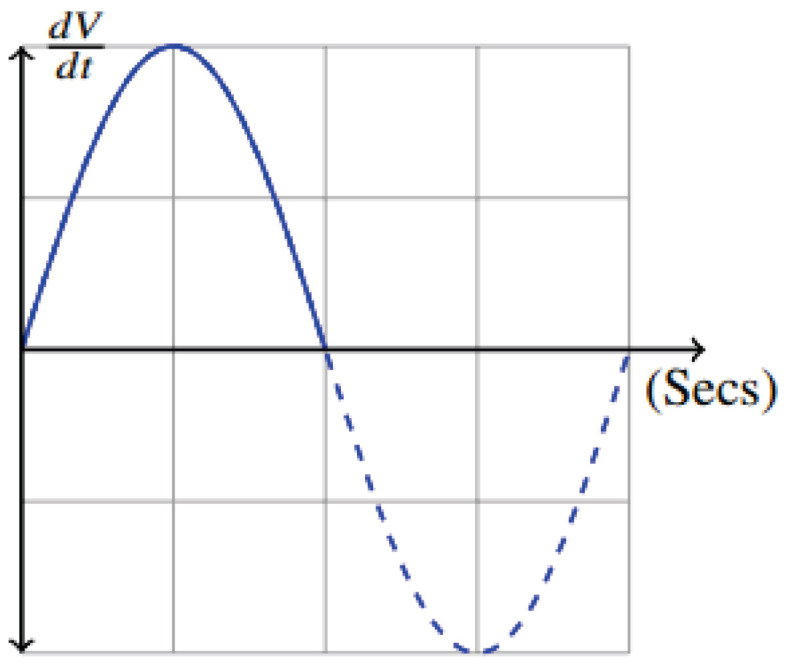
Idealized representation of inspired instantaneous flow as sine wave utilizing a pneumotachogram.

To avoid these invasive transducers, airflow models have been developed to estimate the total internal WoB in a non-invasive fashion [22,23]. Besides avoiding invasive measurements, the airflow WoB model is a great mathematical tool that can be used to run simulations of hypothetical breathing patterns and assess the WoB of these patterns. The airflow WoB model that is widely known is the one developed by Otis and colleagues. This model divides WoB into three forces: elastic, viscous, and turbulent. The elastic work includes the work undertaken to overcome the elastic recoil of the lung and the work undertaken to overcome the elastic recoil of the chest. The viscous work encompasses the work undertaken to overcome tissue resistance, including chest wall and lung resistance. Finally, turbulent work involves overcoming airway resistance, which includes both the airways’ resistance and the resistance of airway devices and circuits. Viscous and turbulent work are often conflated in the pressure–volume loop and referred to as resistive work. Otis’ initial assumptions are that breathing is a pure sinusoidal process (where your time of inspiration is equal to your time of expiration), and work is generated only for the inspiratory phase of the breath (expiration is passive). Thus, this WoB airflow model examines only the instantaneous inspiratory flow of a single cycle’s breath of a pure sinusoidal wave,

(1) $$\frac{dV}{dt}=a\cdot sin\left(2\pi ft\right)$$

where *a* is the amplitude, *f* is frequency, and *t* is time. This velocity pattern of the instantaneous flow of the inspired breath, $\frac{dV}{dt}$, in Equation (2) can be visually shown in Figure 1.

The integration of Equation (1) allows us to define the tidal volume, $V_{T}$,

(2) $$\;V_{T}=\int_{0}^{\pi }a\cdot sin\left(2\pi ft\right)dt.$$

**Figure 2:**
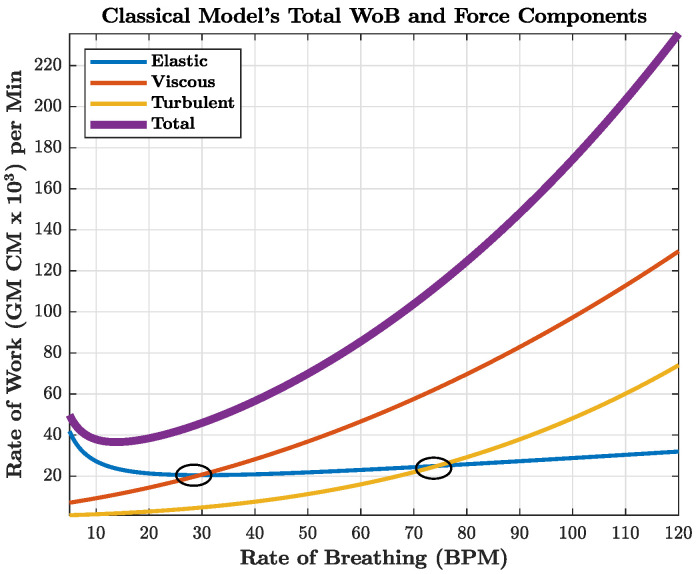
Breakdown of WoB forces using the Classical Sinusoidal Model. The overall internal WoB is composed of three key components: (1) Elastic work, which includes the effort required to overcome the elastic recoil of both the lungs and chest wall; (2) Viscous work, which accounts for the resistance posed by tissues, including lung and chest wall resistance; and (3) Turbulent work, which encompasses the effort needed to overcome airway resistance, including both intrinsic airway resistance and additional resistance from airway devices and circuits. The circles in the figure show where the viscous and turbulent forces overtake the elastic forces.

Thus the final equation that quantifies the rate of inspiratory work undertaken in a minute is defined as

(3) $$\begin{array}{c} W=\frac{1}{2}KfV_{T}^{2}+\frac{1}{4}K^{\prime }\pi^{2}{\left(fV_{T}\right)}^{2}+\frac{2}{3}K^{\prime \prime }\pi^{2}{\left(fV_{T}\right)}^{3}, \end{array}$$

where $V_{T}$ is the tidal volume, *f* is the inspiratory frequency in breaths per minute, and *K*, $K^{\prime }$, and $K^{\prime \prime }$ are the estimated coefficients of resistance for the elastic, viscous, and turbulent forces, respectively [23,24]. The coefficients (*K*, $K^{\prime }$, $K^{\prime \prime }$) were calculated by experiments from Otis [23]. As shown in Equation (3) and Figure 2, it is worth noting that the elastic forces only increase as a function of frequency linearly, but the turbulent and viscous forces increase exponentially as a function of frequency by the second and third power, respectively. The circles within Figure 2 of the classical model’s breathing rate, otherwise denoted as frequency, show where the viscous and turbulent forces overtake the elastic forces.

#### Key Mathematical Take-Away

**Figure 3:**
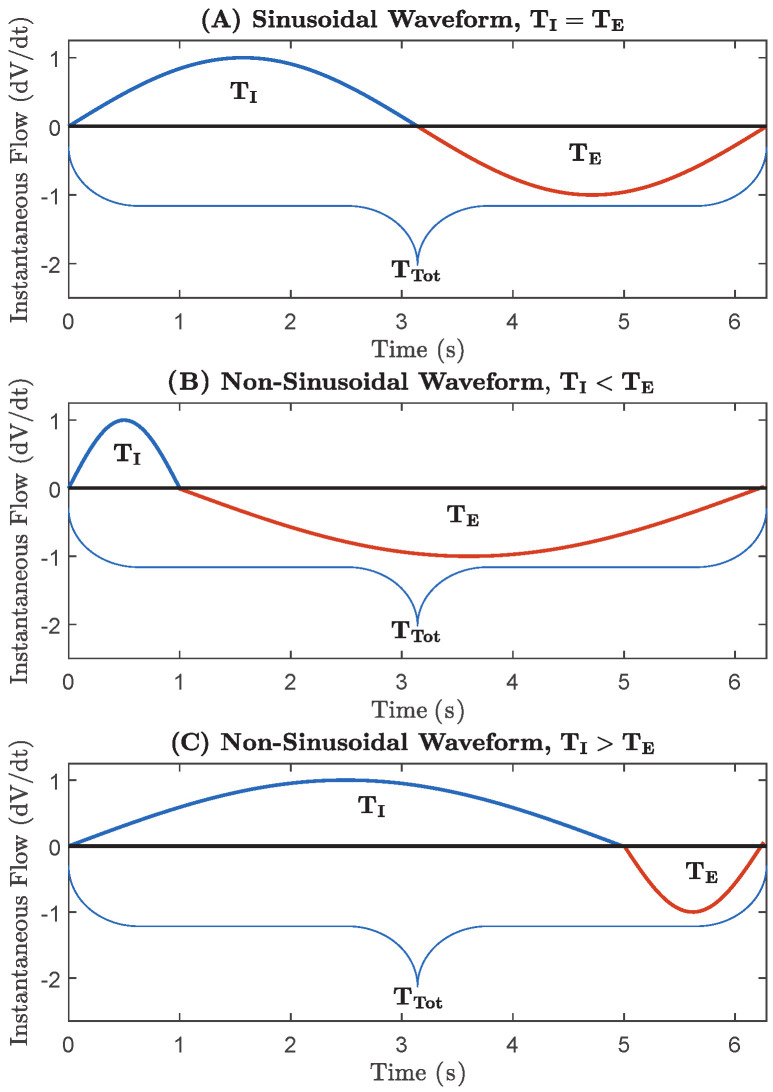
A pictorial example of different waveforms. A waveform is considered sinusoidal if $T_{I}=T_{E}$ for a singular breath. Non-sinusoidal waveforms consider $T_{I}$ and $T_{E}$ dynamic, and examples of these dynamics are conveyed in (graphs **A**–**C**).

The pivotal takeaway without becoming overwhelmed by the mathematics is that, within Equations (1)–(3), *f* is the same variable denoted for frequency. This means that the inspiratory frequency, *f*, only evaluates the velocity pattern of the instantaneous flow of the inspiratory phase, which is half a cycle within a breath (this *f* can be denoted as $f_{i}$, the inspired frequency, or $T_{i}$, the inspired period of the breath). Therefore, the mathematical term frequency, *f*, is used in Equations (1)–(3) to calculate WoB and highlight that

(4) $$\begin{array}{c} f=\frac{1}{2\cdot T_{I}}=f_{i}, \end{array}$$

where $T_{I}$ is the period of inhalation and $f_{i}$ is the instantaneous inspired frequency. The term *f* from the current model assumes that breathing rate and frequency of the breath are interchangeable and $T_{I}=T_{E}$ [22,25]. Therefore, it is paramount that we highlight the important distinction that we often do not breathe with such consistency ($T_{I}=T_{E}$) but rather non-sinusoidally ($T_{I}\neq T_{E}$). This is demonstrated in Figure 3, which depicts non-sinusoidal waveforms within a single breath having unequal inhalation and exhalation phases, pointing out the variability of each breath. This variability caused by non-sinusoidal breathing means that the breathing rate, $B_{R}$, or the number of breaths per minute, is actually not equivalent to *f*. However, breathing rate could be evaluated and interchanged with frequency if the respiratory flow was sinusoidal. This means that the gold standard Otis model uses frequency and breathing rate synonymously in their WoB analysis. Thus, this model formulates WoB for one "perfectly symmetrical breath cycle" (e.g., where total inspiratory, $T_{I}$, and expiratory, $T_{E}$, times are equal) [22,23], as shown in Figure 3A. Due to the fact that these terms of frequency (*f*) and breathing rate ($B_{R}$) are conflated, the classical model utilizes the breathing rate for its analysis of WoB, defined as

(5) $$\begin{array}{c} W_{O}=\frac{1}{2}KB_{R}V_{T}^{2}+\frac{1}{4}K^{\prime }\pi^{2}{\left(B_{R}V_{T}\right)}^{2}+\frac{2}{3}K^{\prime \prime }\pi^{2}{\left(B_{R}V_{T}\right)}^{3}. \end{array}$$

However, we can note that, within Figure 3B, the breathing rate stayed the same, but the inspiratory frequency increased, causing an escalation in work. Comparatively, Figure 3C indicates that the breathing rate remains unchanged, but the inspiratory frequency decreased, causing an attenuation in the internal WoB. Additional pictorial examples of other variations in these non-sinusoidal breathing processes (under the premise that inspiratory frequency is the same but breathing rate changes) have been further highlighted within [22]. Therefore, it is clear that conflating breathing rate and frequency and treating them synonymously does not accurately capture the appropriate work undertaken during a minute of breathing. Thus, no clear model has been proposed to accurately depict WoB [22], and there are fundamental issues on how models neglect frequency and rate information [26]. In normal resting breathing, the failure to differentiate between inspiratory frequency and breathing rate is not a significant issue because inspiratory frequencies are typically low, and elastic forces dominate over viscous and turbulent forces, resulting in minimal errors in airflow models. However, in situations wherein inspiratory frequency increases, such as during strenuous exercise, viscous and turbulent forces become significant, leading to increased errors in current airflow models.

In aviation environments, where AGSM protocols are applied, pilots inhale at frequencies surpassing 2 Hz to prevent dyspnea during high +G_z_ maneuvers [1,7]. These high frequencies drive an exponential increase in WoB, which can cause issues of attentional performance and be linked to aerospace physiological episodes and overall performance [6,27].

Another factor that impacts WoB in aviation is the use of flight masks. These masks create a pressurized and oxygenated environment for the pilot, ensuring proper gas exchange (alveolar ventilation, ${\dot{V}}_{A}$) at high altitudes where oxygen levels are low. The addition of the mask increases the total dead space ($V_{D}$), which includes both physiological dead space ($V_{D_{P}}$) and the mask’s dead space ($V_{D_{M}}$), expressed as ($V_{D}=V_{D_{P}}+V_{D_{M}}$). In this manuscript, any changes in dead space discussed are attributed solely to changes in the mask design ($V_{D_{M}}$), with the physiological dead space ($V_{D_{P}}$) assumed to be fixed at 200 mL.

Thus, it can be noted in Equation (5) that, as $V_{D}$ increases, it removes the available tidal volume, forcing the pilot to increase their breathing rate by

(6) $$\begin{array}{cc} V_{T}-V_{D}= & \;\frac{{\dot{V}}_{A}}{B_{R}}, \end{array}$$

(7) $$\begin{array}{cc} V_{T}-\left(V_{D_{P}}+V_{D_{M}}\right)= & \;\frac{{\dot{V}}_{A}}{B_{R}}. \end{array}$$

Consequently, the use of flight oxygen masks, wherein the air is not participating in gas exchange, increases a pilot’s dead space levels [28]. These additional systems and training techniques, such as oxygen equipment and anti-G maneuvers, are critical and absolutely necessary to implement [29].

We are met with the constraints of utilizing AGSMs and flight oxygen masks to improve the performance of the aviator, but it is also known that these constraints impact the WoB. However, the current WoB models are inadequate for use during high-altitude aerobatic flights wherein flight oxygen masks and AGSMs are required. In order to properly weigh these trade-offs to develop new, improved aviation life-support or training paradigms, it is necessary to ask two fundamental questions which are addressed in this paper: (1) How is WoB impacted during exercises (e.g., AGSM) that require high-frequency inspiratory breathing? and (2) What are the combined impacts of dead space and frequency changes on WoB in high-altitude, masked scenarios?

### 1.3. Challenges

Accurate and precise WoB measurements that include resistive forces (viscous and turbulent forces) are impossible to measure without elaborate novel equipment and strict experimental designs, which require invasive respiratory pressure measurements, as previously discussed in Section 1.2. The widely accepted alternative to this issue is the airflow WoB model, which currently relies on simple sinusoidal models [22,23]. As a result, our approach to breathing analysis and in-flight life-supporting technology is lagging behind the physiological stresses imposed on the pilot in order to operate optimally. However, aviators are trained to increase their ability to withstand gravitational forces and air limitations [9,30]. These current training paradigms, life-support countermeasures, and fundamental understanding of the human–physiological machine interaction and analysis methods are still not adequate, wherein physiological episodes in flight are nevertheless persistent (clear unknowns have not been fully illuminated). Thus, it is clear that the development of these machines (aeronautical airframes) has well surpassed a human’s capability and our complete understanding of how humans physiologically interact with these machines.

These could explain why high-frequency breathing may not have been considered within the sinusoidal model. Similarly, while changes in dead space have been studied previously in [31], the combination of increased dead space at high-frequency breathing has been assumed to be a less significant issue than previously considered. Both of our research questions address the gap between perceived breathing difficulty and actual WoB measurements [32,33,34]. Through our research, we determine where this gap in perception and measurement originates and how we can better model breathing to bridge this gap.

There are numerous factors that drive the perception of breathing. However, enhancing our understanding of accurately measuring the mechanical components associated with the internal WoB will assist our understanding in accurately relating elastic, viscous, and turbulent forces to the sensory feedback loop [35].

### 1.4. Insights

Recently, Napoli and colleagues [22] developed an airflow WoB model for non-sinusoidal breathing that expanded and updated the classical airflow WoB model to account for fluctuating $T_{I}$ and $T_{E}$ [22,23]. This updated model WoB equation is defined by

(8) $$\begin{array}{c} W_{N}=\frac{1}{2}KB_{R}V_{T}^{2}+\frac{1}{4}K^{\prime }\pi^{2}B_{R}f_{I}V_{T}^{2}+\frac{2}{3}K^{\prime \prime }\pi^{2}B_{R}f_{I}^{2}V_{T}^{3}, \end{array}$$

where $B_{R}$ is the breathing rate, $f_{I}$ is strictly the inspiratory frequency, and Otis’s K constants are utilized. This model aims to improve approximated WoB by separating inspiratory frequency and breathing rate terms ($B_{R}\neq f_{I}$), which becomes particularly important in unique aerodynamic contexts. The main differentiation between the two models is that Otis’ model is strictly for sinusoidal breathing dynamics, whereas Napoli’s model focuses on non-sinusoidal breathing and allows for expanded breathing to be explored. Thus, using Equation (8), we can develop models that target aviators’ breathing patterns, especially during AGSMs. By extrapolating these model variables and perturbations, we can develop more accurate estimates of the effects of equipment and techniques. Based upon the extrapolation of breathing rate, inspiratory frequency, and mask dead space ($V_{D_{M}}$) variable ranges outside what has been previously explored by [22,23], we are able to denote changes in dominating forces and implicate better model approximations of breathing in aerospace environments.

### 1.5. Contributions

This paper uses simulated breathing patterns to reveal the impact that high inspiratory frequencies that occur during AGSM breathing have in the WoB by using the airflow WoB model. This complex problem has been overlooked due to the assumptions of sinusoidal breathing patterns, the difficulty of measuring all the breathing forces, and the rare environmental conditions that require unique breathing patterns. These models explain how extreme environments, such as those in aviation, impact how hard pilots work to breathe. The innovative research questions (RQs) that are addressed in this paper provide the following contributions:

RQ1 We discover a critical interaction that drives WoB breathing to extremely high levels that have not been previously reported by clearly delineating respiratory breathing rate and inspiratory frequency. This work shows the importance of the fact that high inspiratory frequency and low breathing rates create significantly higher amounts of work (200–400%) than originally conceived within these extreme environmental cases. This underestimation is driven by how the classical model does not account for inspiratory frequency correctly to attribute the increase in the importance of the viscous and turbulent forces dominating the total WoB equation compared to the elastic forces.
RQ2 Contrary to the current teachings, we demonstrate that breathing rate does not necessarily translate into a significant increase in work as dead space increases and tidal volume is maintained. However, the factor of inspiratory frequency, which is rarely analyzed, creates a much more significant translation into increasing the work of breathing.

## 2. Materials and Methods

Previous airflow WoB models of breathing do not account for high inspiratory frequencies nor the equipment challenges used by aviators during mid-flight, aeronautical maneuvers, or when completing the AGSM. Thus, our investigation into the perturbation of the non-sinusoidal model enables us to reveal the plausibility of respiratory mechanics of high inspiratory frequency breathing conditions and the internal WoB undertaken. To address this gap, we first assess the impact of using the non-sinusoidal model to map these extreme inspiratory frequencies that would be present during flight. Then, we account for changes in WoB as a result of combining high inspiratory frequencies and flight oxygen masks using the appropriate non-sinusoidal instantaneous flow modeling, utilizing Matlab to simulate the physiological processes of interest.

### 2.1. Setting Parameters for WoB Flow Models

We utilized two different types of instantaneous flow WoB models: the foundational sinusoidal (Equation (5)) and the non-sinusoidal model (Equation (8)). Across both models, we maintained the same foundational coefficients, where $K=8.52{\mathrm{cmH}}_{2}$O/L, $K^{\prime }=3.5{\mathrm{cmH}}_{2}$O/(L/s), and $K^{\prime \prime }=1.5{\mathrm{cmH}}_{2}$O/(L/s)^2^ for their respective elastic, viscous, and turbulent force properties [23]. The WoB model incorporates two dead space parameters, the physiological dead space ($V_{D_{A}}$) and the mask’s dead space ($V_{D_{M}}$) that define the total dead space, ($V_{D}$). The physiological dead space was set at 200 mL, and two alveolar ventilation values were explored: one simulating normal resting breathing at 6 L/min and one simulating breathing during an AGSM at 30 L/min. These values were chosen based on the previous literature [7,23,28,36]. To ensure that the alveolar volume is within an acceptable range, we only considered alveolar volume above 0.3 L, which cuts off the breathing rate at 20 BPM for ${\dot{V}}_{A}$ = 6 L/min. The mask’s dead space ($V_{D_{M}}$) is set to zero for the first RQ1 and then later perturbated to address RQ2, which is discussed in Section 2.2.

Although the previous parameters were utilized for both models, we were forced to have deviates between the breathing rate and instantaneous inspiratory frequency between the two models. For the sinusoidal model, we only varied $B_{R}$ between 10 and 60 breaths per minute because $B_{R}=f$. This translates into a max inspiratory frequency of 1 Hz (60 breaths per minute). For the non-sinusoidal model, we varied breathing rate and frequency. The inspiratory frequency (*f*) ranges between 0.25 and 2 Hz and the breathing rate shared the same range as the sinusoidal model of 10 and 60 breaths per minute, when possible. This allowed us to accurately model AGSM breathing techniques that require fast inspiratory breaths. These ranges of frequencies were determined by including both normal ranges of inspiratory frequencies (e.g., those less than 1 Hz) [23] and AGSM breathing ranges up to 2 Hz in brief, instantaneous inspiratory bouts of breathing [7,37].

#### Unit Conversions for WoB Equations

Each resistive coefficient has different units that are associated with the elastic ($W_{E}$), viscous ($W_{V}$), and turbulent forces ($W_{T}$). The unit cancellation slightly differs between the non-sinusoidal ($W_{V_{N}}$, $W_{T_{N}}$) and classical model ($W_{V_{C}}$, $W_{T_{C}}$). Therefore, we must take additional consideration that may not be obvious in these calculations by ensuring proper units within the WoB equation to calculate total work ($W=W_{E}+W_{V}+W_{T}$) for both types of models. To do this, conversions for $K^{\prime }$, $K^{\prime \prime }$, *f*, ${\dot{V}}_{A}$, and $V_{D}$ were included to provide an expansion of units for proper calculation for each of the WoB components for the non-sinusoidal and classical models. These variable units are as follows: $K=8.52{\mathrm{cmH}}_{2}$O/L, $K^{\prime }=3.5{\mathrm{cmH}}_{2}$O/(L/s), $K^{\prime \prime }=1.5{\mathrm{cmH}}_{2}$O/(L/s)^2^, *f* is Hz (also expressed as 1/s), $B_{R}$ is BPM (also expressed as Breaths per Min), ${\dot{V}}_{A}$ is L/min (also expressed as L·(Breaths per Min)), and $V_{D}$ is in liters (L). These changes resulted in the rate of work (power) to be measured in (GM-CM × $10^{3}$)/min, where GM-CM is a unit of work in the cgs system equal to the work undertaken when raising a weight of one gram against the force of gravity to a height of one centimeter. These internal work-of-breathing units can be translated into typical life support systems that impose external WoB, which utilize joules per liter (1 GM-CM = 0.000098 J).

We provide an example numerical solution for the reader to verify their understanding using the assumed values to equal ${\dot{V}}_{A}$ = 6 L/min, $V_{D}=0.2$ L, $B_{R}=15$, and $f_{I}=0.45$. Therefore, we arranged the following WoB forces components for unit cancellation as follows:

***Tidal Volume***

(9) $$\begin{array}{cc} V_{T} & =\frac{{\dot{V}}_{A}}{B_{R}}+V_{D}=\frac{{\dot{V}}_{A}\overset{{\dot{V}}_{A}\;w/units}{\overbrace{\left(\frac{L}{\min}\right)}}}{B_{R}\overset{B_{R}\;w/units}{\overbrace{\left(\frac{1}{\min}\right)}}}+V_{D}\overset{V_{D}\;w/units}{\overbrace{\left(L\right)}} \end{array}$$

This demonstrates the unit cancellation process for tidal volume as a function of alveolar ventilation (${\dot{V}}_{A}$), dead space ($V_{D}$), and breathing rate ($B_{R}$). Therefore, $V_{T}$ will strictly use the units of liters (L) of the following calculations for elastic, viscous, and turbulent, which will be expressed as L, $L^{2}$, and $L^{3}$, respectively.

***Elastic Forces:*** (Classical and Non-Sinusoidal Model).

(10) $$\begin{array}{cc} W_{E}= & \frac{1}{2}K\cdot B_{R}\cdot V_{T}^{2}\\ = & \frac{1}{2}K\cdot B_{R}\cdot V_{T}\left(\frac{cm\;H_{2}O\cdot L}{Mins}\right)\\ = & 23.004\left(\frac{cm\;H_{2}O\cdot L}{Mins}\right) \end{array}$$

***Viscous Forces:*** (Classical Model).

(11) WVC=14K′·π2·BR2·VT2=14K′cm·H2O·SL⏞K′withUnits·π2·BR21Mins2⏞BRwithUnits·VTL2⏞VTwithUnits=14K′cmH2O·S1·π2·BR21Mins·160S·VTL=14K′·π2·BR2·160·VTcmH2O·LMins=11.659cmH2O·LMins

***Viscous Forces:*** (Non-Sinusoidal Model).

(12) WVN=14K′·π2·BR·f·VT2=14K′cmH2O·SL⏞K′w/Units·π2·BR1Mins⏞BRw/Units·f1S⏞fw/Units·VTL2⏞VTw/Units=14K′·π2·BR·f·VTcmH2O·LMins=20.986cmH2O·LMins

***Turbulent Forces:*** (Classical Model).

(13) WTC=23K″·π2·BR3·VT3=23K″cmH2O·S2L2⏞K″w/Units·π2·BR31Mins3⏞BRw/Units·VTL3⏞VTw/Units=23K″cmH2O·S21·π2·BR31Mins·13600S2·VTL=23K″·π2·BR3·13600·VTcmH2O·LMins=1.999cmH2O·LMins

***Turbulent Forces:*** (Non-Sinusoidal Model).

(14) WTN=23K″·π2·BR·f2·VT3=23K″cmH2O·S2L2⏞K″w/Units·π2·BR1Mins⏞BRw/Units·f21S2⏞fw/Units·VTL3⏞VTw/Units=23K″·π2·BR·f2·VTcmH2O·LMins=6.475cmH2O·LMins

### 2.2. Non-Sinusoidal Modeling of Dead Space Changes During Dynamic Flight Environments

We then used the non-sinusoidal equation (Equations (7) and (8)) to model the combined effects of frequency and dead space imposed by a flight oxygen mask. We accomplished this by calculating changes in breathing rate and a flight oxygen mask’s dead space while holding all other variables constant. The mask’s dead space values were set at a range from 100 to 300 mL to assess critical levels for a mask’s design (low (100 mL), normal (200 mL), and high (300 mL)) [28]. Thus, 100 mL of dead space for a flight oxygen mask would be a practical design, as opposed to a flight oxygen mask that creates 300 mL of dead space which would be a poor design. The physiological dead space criteria was set at 200 mL. We conducted the analysis four times. We simulated the non-periodic breathing patterns using breathing rate ranges from 10 to 60 BPM and inspiratory frequencies from 0.25 to 2 Hz with 0.25 Hz step increments. This enables us to expand work previously completed by [22] to account for the elevated frequencies above 1 Hz and dead space changes for pilot breathing environments. Using these parameters, we are able to draw conclusions about the combined effects of the model by comparing our resulting figures.

### 2.3. WoB Comparison Across Models

In order to evaluate how the classical model compares to the non-sinusoidal model, we provide a percent change calculation, $P_{C}$. This is calculated using Equation (5), the classical model’s sinusoidal approach ($W_{O}$), and Equation (8), the nascent non-sinusoidal model ($W_{N}$) calculations, to be inputted into equation

(15) $$P_{C}=\frac{W_{N}-W_{O}}{W_{O}}*100.$$

Therefore, if $P_{C}$ is positive, it indicates that the non-sinusoidal model ($W_{N}$) produces higher values compared to the classical model ($W_{O}$), meaning that the classical model underestimates the work per min. Whereas if $P_{C}$ is negative, it indicates that the classical model overestimates the work per min. This mismatch between the two models is a function of high inspiratory frequency and avoids the assumption of non-sinusoidal breathing, which is not accounted for in the classical model.

## 3. Results

By deploying the two WoB equations to in-flight contexts, we can demonstrate how much the internal WoB can be underestimated in aviators. This allows us to specifically address our two proposed research questions, RQ1 and RQ2. The first RQ1 assesses the differences between the classical and non-sinusoidal models as a function of breathing rate and inspiratory frequency for normal breathing and those used during AGSM breathing (high inspiratory frequency). Our second research question, RQ2, highlights how WoB is impacted by masked environments for high-performance aircraft when the non-sinusoidal models are adjusted for flight oxygen-masked dead space and inspiratory frequency. Using these models, we show how sinusoidal models can greatly underestimate an aviator’s total WoB, while non-sinusoidal models potentially provide new insight into the specific equipment and techniques of a pilot’s breathing effort.

### 3.1. RQ1: High-Frequency Inspiratory Breathing

#### 3.1.1. Analysis of Percent Change in Total Internal WoB Across Models

**Figure 4:**
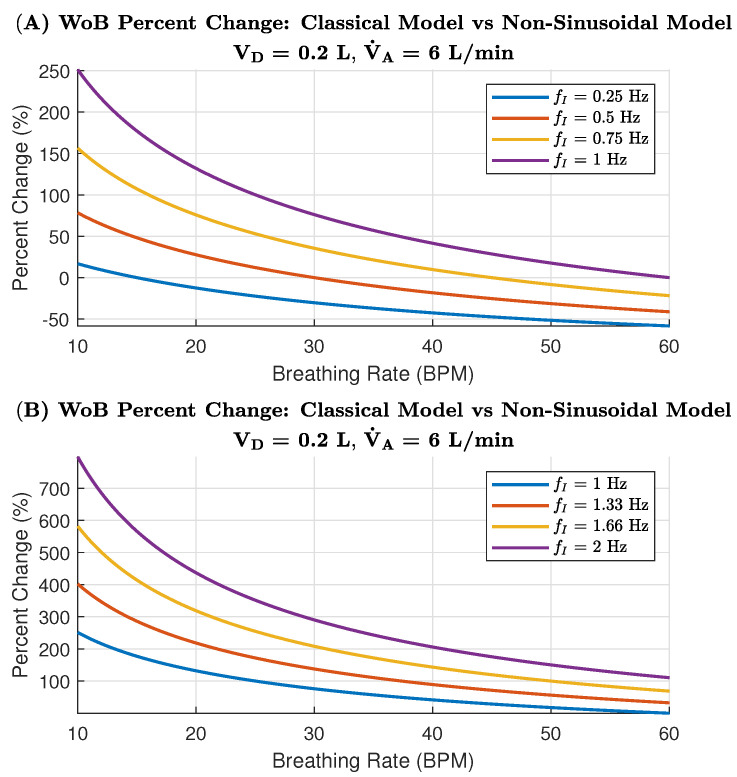
Percentage change in WoB between the Classical Sinusoidal Model and the Non-Sinusoidal Model for both low and high inspiratory flows, considering an alveolar ventilation of 6 L/min. A positive percentage change indicates that the Non-Sinusoidal Model results in a higher WoB compared to the Classical Model.

The percent change in WoB between sinusoidal and non-sinusoidal models at ${\dot{V}}_{A}$ = 6 L/min is demonstrated for lower inspiratory frequencies in Figure 4A and higher inspiratory frequencies in Figure 4B across a range of breathing rates. The lower inspiratory frequencies in Figure 4A show a gradual decrease in percent change as the breathing rate increases. Specifically, for frequencies of 0.25 Hz, 0.5 Hz, 0.75 Hz, and 1 Hz, the percent change in WoB decreases steadily from 10 to 20 breaths per minute. The higher inspiratory frequencies in Figure 4B similarly demonstrate a decrease in percent change with increasing breathing rates. Frequencies of 1 Hz, 1.33 Hz, 1.66 Hz, and 2 Hz all show this trend, with the highest percent change at lower breathing rates and the lowest change at higher breathing rates. This pattern indicates that, at lower breathing rates, the discrepancy between the classical and non-sinusoidal models is greater, especially at higher inspiratory frequencies.

This is particularly relevant for scenarios such as the AGSM breathing protocol, wherein low breathing rates combined with high inspiratory frequencies result in a marked difference in the assumed WoB. When examining relatively normal respiration rates, around 12–16 BPM [38] and fixing a specific breathing rate (i.e., 15 BPM), we can note that the WOB drastically changes when the AGSM inspiratory frequencies are modeled in the total internal WoB equation. Figure 4B’s indicates an AGSM breathing rate of 15 BPM, which suggests an increase change in WoB from 175% and up to 550%. Thus, these aviators are operating using a significantly higher WoB than previously thought when using the classical model.

**Figure 5:**
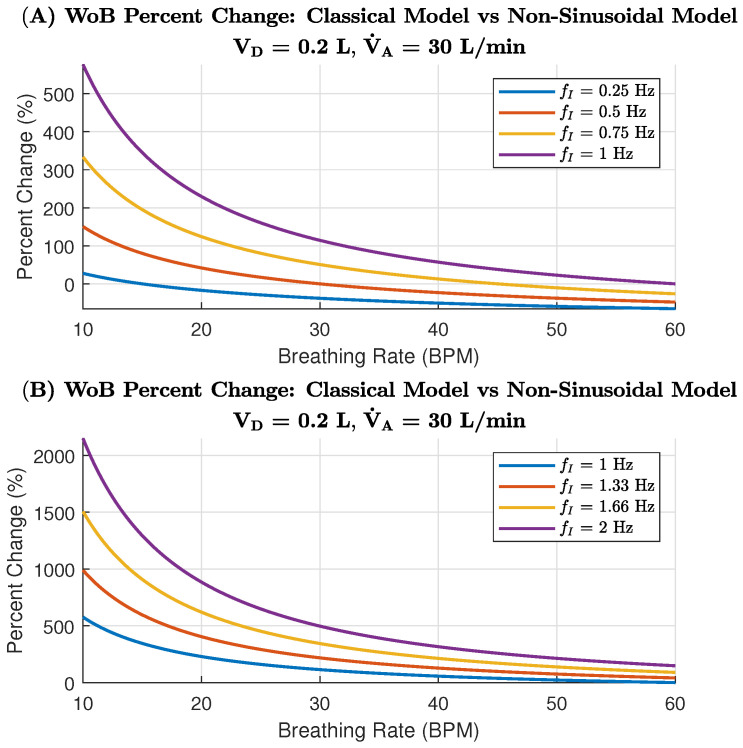
Percentage change in WoB between the Classical Sinusoidal Model and the Non-Sinusoidal Model for both low and high inspiratory flows, considering an alveolar ventilation of 30 L/min. A positive percentage change indicates that the Non-Sinusoidal Model results in a higher WoB compared to the Classical Model.

The second simulated WoB model was explored for an alveolar ventilation of 30 L/min, showing the difference in WoB rate between sinusoidal and non-sinusoidal models. Similar to the 6 L/min model (Figure 4A), the lower inspiratory frequencies in Figure 5A show a clear decrease in percent change as the breathing rate increases. However, it is important to note that the rate of change for ${\dot{V}}_{A}$ = 30 L/min is much higher than for ${\dot{V}}_{A}$ = 6 L/min. Thus, as alveolar ventilation increases, the classical model further overestimates the WoB. However, as the breathing rate increases, the two models converge more quickly, arriving at a closer estimated agreement on the WoB.

#### 3.1.2. WoB Breakdown: Elastic, Viscous and Turbulent Forces

**Figure 6:**
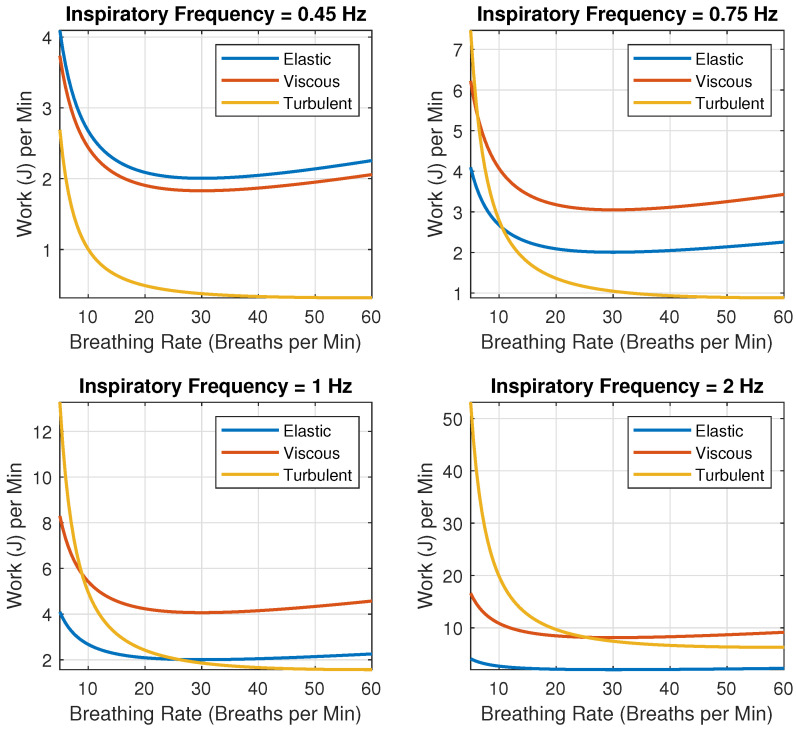
Elastic, Viscous and Turbulent Force breakdown for various inspiratory frequencies for an alveolar ventilation of 6 L/min model.

The classical and non-sinusoidal alveolar ventilation 6 L/min model comparison conveys the importance of recognizing the differences in the models based on inspiratory frequency and breathing rate. To better understand which forces contribute to the overall WoB result, we break down the forces included in the equation and assess them at varying inspiratory frequencies and rates in Figure 6. Across all inspiratory frequencies, the components of the WoB are the greatest at lower breathing rates, approximately below 20 BPM. However, as inspiratory frequency increases, the viscous and turbulent components for WoB increase more dramatically than the elastic component (Seen in Equation (8)). For example, when an inspiratory frequency of 0.45 Hz is presented in Figure 6, the elastic forces are clearly the most dominant in determining overall WoB. However, as the frequency increases from 0.45 to 0.75 and then to 1 Hz in Figure 6, the elastic forces drop in their overall total contribution as the viscous and turbulent forces begin to be the dominant contributors for the WoB. At the final inspiratory frequency of 2 Hz, the elastic forces are negligible in determining the overall WoB. This overall overtaking of elastic forces by the viscous and turbulent forces begins around 0.5 Hz. The results indicate that a pilot’s WoB during AGSMs is significantly impacted by non-sinusoidal respiratory models, especially at higher inspiratory frequencies and lower breathing rates. Compared to sinusoidal models, the non-sinusoidal models show a marked increase in WoB, with discrepancies as high as 550% at typical AGSM breathing rates (15 BPM). This increase is driven by a greater contribution from viscous and turbulent forces as the inspiratory frequency rises, suggesting that classical models underestimate the actual WoB experienced by pilots during AGSMs. When expanding the model to higher alveolar ventilation (e.g., 30 L/min), although we can note all the forces increasing, the frequencies at which the forces overtake one another are altered. Thus, how these forces interact and overtake each other is a function of alveolar ventilation. For the case of 30 L/min alveolar ventilation, the turbulent forces completely overtake the viscous and elastic forces at all frequencies of 0.45, 0.75, 1.0, and 2.0 Hz. In this scenario, the elastic force maintains dominance over the viscous force until 0.5 Hz. Therefore, the dominance between the elastic and viscous forces appears less sensitive to changes in alveolar ventilation.

### 3.2. RQ2: Impacts of Frequency and Dead Space on WoB

**Figure 7:**
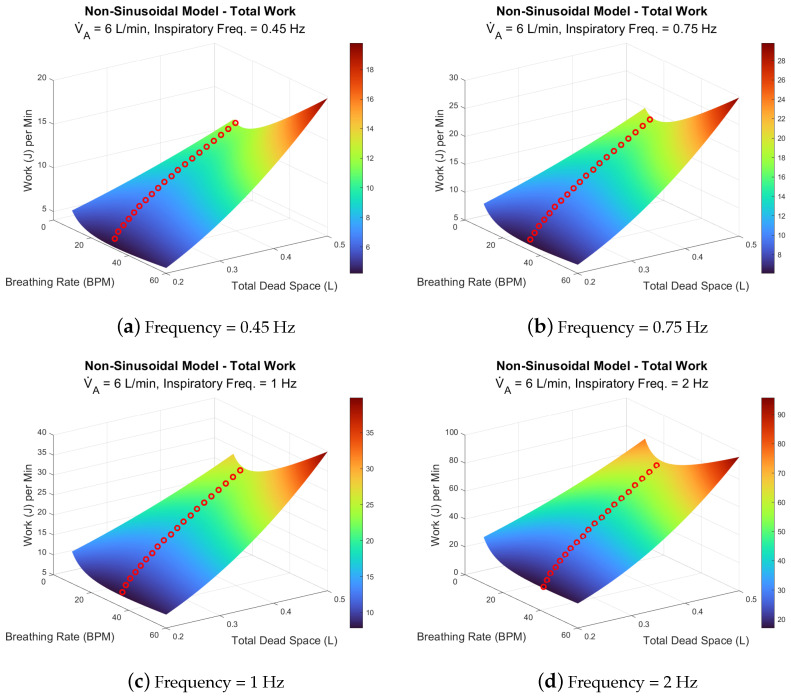
Frequency and dead space effects on WoB at resting alveolar ventilation (6 L/min). The red circles indicate the optimal breathing rate for a given dead space.

**Figure 8:**
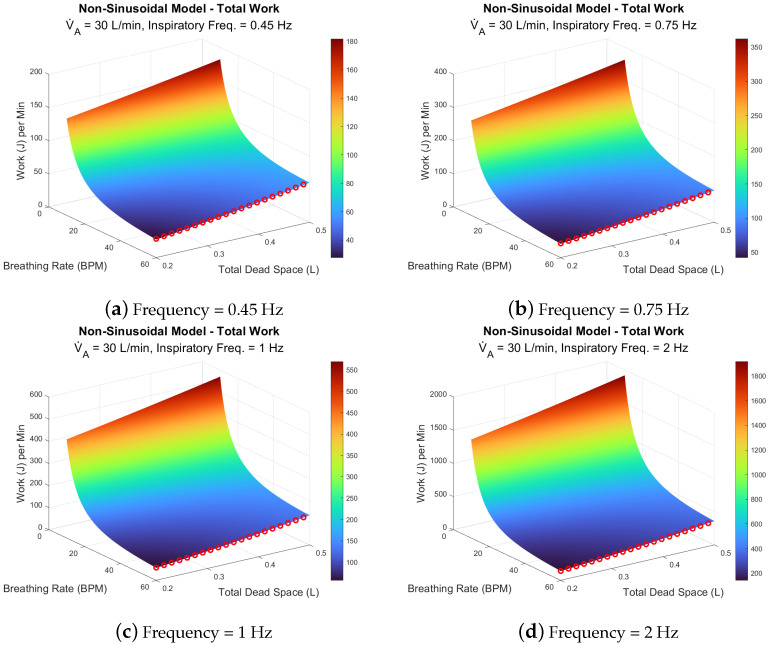
Frequency and dead space effects on WoB at high alveolar ventilation (30 L/min). The red circles indicate the optimal breathing rate for a given dead space.

Using Equations (7) and (8), we calculated the WoB rate at various breathing rates, dead space volumes, and inspiratory frequencies. The dead space volumes ranged from 0.2 to 0.5 L, while the inspiratory frequencies were 0.45, 0.75, 1, and 2 Hz. This is presented across two different alveolar ventilation rates, 6 L/min and 30 L/min, as shown in Figure 7 and Figure 8, respectively.

Regarding the first model with an alveolar ventilation of 6 L/min, Figure 7a–d shows how the WoB rate varies according to changes in breathing rate, inspiratory frequency, and dead space. As total dead space increases, WoB generally rises, with more pronounced effects observed at both lower and higher breathing rates. These significant changes in dead space illustrate how the surface area of work is concave, where the optimal breathing rate is central rather than at the edges of the surface. At a lower total dead space volume (0.1 L), the WoB rate remains relatively unaffected across breathing rates, with a minimum work rate observed around 30–35 BPM. The optimal breathing rate, indicated by red circles, shifts depending on the dead space volume, revealing that for a given dead space, there is a specific breathing rate that minimizes the total WoB. As dead space increases, a pilot’s breathing rate must decrease to achieve a new minimum WoB. This shift in the minimum is also influenced by inspiratory frequency, as seen in Figure 7a,d, where the minimum shifts to higher breathing rates as inspiratory frequency increases.

The second model with an alveolar ventilation of 30 L/min is shown in Figure 8a–d. It should be noted that the surface area topology of work across all inspiratory does not alter, where the dead space doesn’t play a significant role in impacting the WoB. Thus, with higher alveolar ventilation rates, WoB is less sensitive to dead space changes, where achieving an optimal minimum WoB is accomplished by increasing your breathing rate. This is illustrated by the red circles highlighting the optimal WoB at the highest breathing rates within Figure 8, which borders to be not physiologically relevant. However, although the topology is relatively the same, as the inspiratory frequency increases, the range of total work values also increases. At 0.45 Hz (Figure 8a), total work ranges from approximately 30 to 180 J/min, while at 2 Hz (Figure 8d), it ranges from around 200 to 1800 J/min. In all conditions, total work steadily increases with lower breathing rates and larger total dead space volumes. The gradient of total work becomes steeper as inspiratory frequency rises, with the largest increases observed at 2 Hz. In contrast, lower frequencies, such as 0.45 and 0.75 Hz, exhibit more moderate increases in total work across the same ranges of breathing rate and dead space.

## 4. Discussion

The key messages within Section 4 highlight that, during AGSMs, the classical airflow WoB models do not properly capture the factors that fully drive WoB, and they only examine breathing rate, leading to disparate results. In fact, by conflating breathing rate and inspiratory frequency, the level of work is drastically underestimated and can lead to a mismatch of perception. This mismatch between the true level of work and perception is confirmed by high perceived effort but reports of only low levels of work in previous models [32,33,34]. Consequently, the purpose of analyzing the WoB under extreme conditions is to lay the foundation for which accurate factors within the respiratory signal need to be analyzed. This will then inform the community on the necessity for specific new analyses in order to develop new accurate WoB tools and their translation to life support systems for aviators. Specifically, our research answered the following research questions: RQ1) How is a pilot’s WoB impacted during AGSMs using non-sinusoidal respiratory models; and RQ2) What are the impacts of dead space on WoB when utilizing novel non-sinusoidal respiratory models?

### 4.1. RQ1: High-Frequency Inspiratory Breathing

Our study reveals significant disparities in the classical WoB model’s ability to accurately represent how hard a pilot works to breathe during elevated inspiratory frequencies, particularly under AGSM protocols. The classical model conflates breathing rate with frequency, assuming pure sinusoidal breathing patterns. By distinguishing these terms, we developed a non-sinusoidal model that better captures the total internal WoB for aviators, especially under non-standard conditions. Our findings, based on RQ1 and analysis of Equation (8), demonstrate that WoB is driven by three primary components: elastic, viscous, and turbulent forces $\left(W=W_{E}+W_{V}+W_{T}\right)$. Importantly, the elastic force depends on breathing rate alone, while the viscous and turbulent forces incorporate frequency terms (*f* and $f^{2}$), making them more influential at higher inspiratory frequencies.

AGSM breathing patterns involve abnormally high inspiratory frequencies (0.5 to 2 Hz) paired with moderately normal breathing rates (12–30 BPM). In extreme cases, pilots may even surpass 2 Hz, reaching up to 4 Hz during maximum inspiratory efforts, as highlighted in previous research [22]. Under such conditions, the elastic forces become negligible, while the resistive components—viscous and turbulent forces—dominate. These forces, which depend on the frequency of breathing, are also influenced by factors such as the viscosity and density of the respiratory gas [23,39,40], tissue properties [22,23], and the structure of the respiratory system (e.g., airway bifurcations [41,42,43]). Understanding these factors is crucial for explaining two key dynamics: (1) the largest percentage change in WoB between models occurs during low breathing rates with high inspiratory flow (Figure 4 and Figure 5); and (2) the increase in viscous and turbulent forces at high inspiratory frequencies contrasts with their reduction at higher breathing rates (Figure 7 and Figure 8).

The classical WoB equation, focused on alveolar ventilation, overlooks the role of inspiratory frequency in determining airflow speed into the lungs. It simplifies the relationship between breathing rate, flow speed, and air volume per breath. In contrast, the non-sinusoidal model distinguishes the rate of airflow and volume intake per breath. For instance, with low breathing rates and high inspiratory frequencies, more air is moved into the lungs per breath at higher speeds, leading to greater lung tissue stretching and increased respiratory gas density. This, in turn, heightens the turbulent and viscous components of WoB, explaining the larger percentage changes between models in low breathing rate and high inspiratory flow scenarios (as seen in Figure 4). Conversely, increasing the breathing rate reduces the viscous and turbulent components, as breaths become shallower, preventing excessive tissue stretch and gas compression, as depicted in Figure 7. Mathematically, the analysis shows that higher breathing rates decrease $V_{T}^{3}$, as the breathing rate is inversely related to tidal volume ($V_{T}$), explaining why WoB is more elevated at lower breathing rates where tidal volumes are larger and result in greater turbulence.

While these findings may seem extreme, they highlight the importance of accurate modeling key factors (e.g., inspiratory frequency) for specific populations, such as aviators who practice breathing at elevated inspiratory frequencies. Although pilots represent a small subset of the population, their unique needs underline the value of understanding and addressing the respiratory challenges they face. Through this improved modeling, we can better anticipate breathing difficulties and optimize support systems for pilots performing AGSM, ultimately contributing to safer and more effective flight operations.

### 4.2. RQ2: Impacts of Frequency and Dead Space on WoB

Our study demonstrates that increased dead space and elevated inspiratory frequencies exponentially heighten the WoB rate, posing a greater risk to aviators than initially anticipated by classical WoB models. This is critical, as pilots often rely on high-frequency breathing for safety and operational effectiveness during flight. The results indicate that the risk of dyspnea becomes particularly pronounced when high inspiratory frequencies are compounded with increased dead space. This finding suggests that life-support engineers and pilots must recognize this increased risk and adapt accordingly.

Interestingly, our study challenges the traditional assumption that lower breathing rates are inherently more efficient. We found that at alveolar ventilation of 6 L/min, higher breathing rates (approximately 35 BPM) can result in a lower total WoB. This lower WoB at higher breathing rates is because, at higher tidal volumes, the WoB increases because of the larger volume of air being inspired. Therefore, our results suggest that taking more frequent but shallower breaths can help improve the WoB, reducing the energy required to move larger air volumes with each breath. This insight suggests that a balanced approach—optimizing both the breathing rate and the inspiratory frequency—can reduce energy expenditure during breathing, even under conditions that typically elevate the WoB.

These findings hold significant implications for pilot training and the design of life-support systems. They indicate that training pilots to optimize their breathing patterns may be possible, thereby reducing WoB and mitigating the risk of dyspnea. This understanding could also inform the design of adaptive life-support systems that adjust to physiological demands during different flight conditions, such as high-G maneuvers. However, our study has limitations, this is an overly simplified respiratory model for such complex environmental and physiological conditions. Future research should aim to validate these results under a broader range of flight scenarios and different breathing gear to enhance generalizability.

Our results motivate exploring the development of training programs that focus on optimizing breathing patterns for energy efficiency, ensuring pilots are better equipped to manage the respiratory challenges they face. Additionally, investigating adaptive life-support systems that respond to real-time physiological data could offer further advancements in maintaining respiratory efficiency during flight. Such research would help bridge the gap between theoretical models and practical applications, ensuring that pilots can make informed decisions during critical situations and improve their performance under challenging conditions.

### 4.3. Strengths and Limitations

The non-sinusoidal WoB model selected for this research permitted simplifications in breathing while also accounting for the important differences between frequency and breathing rate. These fundamental strengths allowed us to draw conclusions about breathing in an aviator’s dynamic environment. However, testing our model with subjects is difficult due to the limitations associated with measuring breathing in humans while in flight. To enhance the practical application of these models, future research should focus on validating these results with in-flight data and pilot experimental testing to corroborate the simulation-based findings. The lack of in-flight data explains why simplified models that measure only elastic forces at the mouth are popular but lead to erroneous results. It is important to emphasize that these coefficients (K, K’, K”) are just estimates of the elastic, viscous, and turbulent resistances and can be subject to change at high altitudes and vary across individuals (e.g., body frame, pulmonary health, anatomy, etc.). At high altitudes, changes in the Reynolds number will occur, impacting flow velocity, the anatomical diameter, and air density [44], which can potentially reduce these coefficients that approximate resistance. Thus, the fundamental insights that emphasize how breathing dynamics change as a function of frequency and breath with respect to WoB will be altered. The importance of how fast the instantaneous flow of air enters the lungs with respect to $V_{T}$ is independent of breathing rate and is critical to understanding WoB, pulmonary damage, atelectasis, and respiratory training. Therefore, the WoB with respect to $V_{T}$ and instantaneous inspiratory flow should potentially change with respect to body frame and body position. Thus, this research may also be expanded to further determine the differences between sexes during breathing (populations with statistically different body frames and $V_{T}$). This is also supported by previous research [39] that observed women having higher WoB due to increased resistive WoB and a higher breathing rate. Women additionally have a lower maximum tidal volume, which is attributed to smaller lungs and less dead space [39]. With this knowledge, it may be concluded that women, or statistically different body frames, complete AGSMs in a different manner to optimize their WoB. It is also important to mention that the current state of this model only accounts for the elastic and resistive forces. The respiratory system is subjected to multiple other forces, such as inertial, gravitational, and distorting forces of the chest wall. During normal resting breathing, these forces are usually neglected, but they become important in extreme contexts such as aviation. Finally, this model only accounts for the inspiratory WoB. If the expiration is not passive, as sometimes might be the case in extreme environments, the expiratory WoB would need to be accounted for as well, as the effort required to actively exhale could significantly underestimate the total WoB in such environments.

## 5. Conclusions

There are several factors that affect airflow in the airways. Airway resistance in the presence of laminar flow is expressed as a function of the Hagen–Poiseuille equation [45]:

(16) $$R=\frac{8\cdot h\cdot l}{\pi \cdot r^{4}},$$

where *h* is the viscosity, *l* is the length, and *r* is the radius.

In airway resistance, the most important factor is the radius of the airway. Very small changes in airway radius result in significant changes in airway resistance. The portion of the respiratory tree with the smallest radius is the medium-sized bronchi. Airway resistance varies in the respiratory cycle between inspiration and expiration. The majority of airways are tethered in the lung by alveolar attachments that pull on the airway, aid in expansion, and keep airways open. This is labeled as radial traction of the airways. Radial traction and lung expansion with inspiration increase the force on the airway outwardly and result in increasing the airway radius, thus decreasing airway resistance. With expiration, the pull on the airway diminishes, and intrinsic airway elastic recoil increases against the radial traction, causing a decrease in airway radius, which leads to increased airway resistance [45].

Under +G_z_ acceleration, the external force of the load is against the chest wall and lungs, forcing the lungs to contract and increasing airway resistance, which can result in turbulent flow. Once turbulent airflow is present, the Hagen–Poiseuille equation is no longer valid and the Darcy–Weisbach equation must be applied:

(17) $$R=f\cdot \frac{L}{D}\cdot \frac{\rho v}{2}$$

where *R* is the resistance due to turbulent flow in the airway, *f* is the Darcy friction factor, a dimensionless number that represents the frictional resistance of the flow within the airway. It depends on the Reynolds number and the relative roughness of the airway. *D* is the diameter of the airway, ρ is the density of the fluid (air in this case), and *v* is the flow velocity of the fluid within the airway (e.g., instantaneous airflow). Furthermore, the muscles taking part in inhalation exert a force against the gravity vector, and this requires a successively greater effort to expand the increasingly heavier chest wall and parenchymal lung tissue. The AGSM has been shown to be the most effective method to counteract the G-induced loss of consciousness [2,4,46,47]. The AGSM is augmented by pressure breathing at maximally 8.0 kPa (60 mmHg). This mask system of incremental pressurization is automatically increased in response to heavier +G_z_ accelerations. With a mask pressurization system, the pilot is able to use less effort to counteract the +G_z_ forces and maintain an expanded airway to inspire [48]. Research has demonstrated that, with higher +G_z_ loads, the inhalatory flow is diminished, resulting in an increase in breathing difficulty [49]. This is proportional to the +G_z_ load and anti-G suit inflation. What is notable is that work appears unaffected by the +G_z_ loads as well as the inflation of the anti-G suit. Thus, the key portion to understand is the inhalation WoB to discern the resistance and effort needed in that portion of the respiratory cycle.

Aviation involves a great amount of risk that can be minimized through knowledge, training, and equipment. Informing pilots of in-flight breathing dynamics could decrease the risk associated with high-altitude flight, allowing the pilots to stay focused and alert during a variety of missions and in-flight situations [50,51]. The results of previous research have improved pilots’ safety and capabilities [52,53]. However, research in the area of breathing has been limited, and a large factor associated with this knowledge gap has been the limitations of the classical WoB equation. This study addresses this gap by introducing novel non-sinusoidal respiratory models, which provide a more accurate assessment of the WoB in dynamic conditions, such as those experienced by pilots during high-G maneuvers. These models, which account for the complex, behavior of airflow and the effects of dead space, represent a significant departure from classical models, offering new insights into respiratory dynamics under extreme conditions. Therefore, it is essential to assess breathing as it relates to aviators to maintain physiological awareness during missions. In addition, clinical solutions such as advanced respiratory muscle training [54], improved flight mask designs to minimize dead space, and real-time physiological monitoring [6] could further mitigate the challenges highlighted in our findings. Based on the information introduced in this paper, it is revealed that WoB levels in the cockpit are more crucial to consider than originally thought.

The findings in this study, while focused on aviation, can be adapted to other fields such as space exploration, underwater activities, and high-performance sports, where understanding and managing respiratory load is equally critical. The novel modeling approaches presented here could be applied to life support systems and training paradigms, providing a new avenue for enhancing human performance in extreme environments. Our understanding of aviators’ breathing dynamics in such environments has applications to life support systems in future space flight applications [4,55,56,57]. Space exploration is one of the most challenging and dangerous endeavors an aviator can embark on. Taking the extreme environment into consideration, careful thought and planning must be placed into the development of equipment and training paradigms to meet the physiological demands of WoB experienced by future explorers [4,57,58,59,60,61]. Life-support systems must be carefully cultivated to meet the various environments and conditions of humans. Additionally, it is possible that the effects of high-G breathing may begin to affect more people than just specialized pilots [2,4,58,61]; advances in space technology for future exploration may result in untrained personnel being exposed to high-G forces during take-off of the space shuttle [2,62,63]. Training may require reconfiguration to meet the diverse needs of differing thoracic cavities undergoing increased gravitational forces to meet the demands of WoB [2,4].

## Data Availability

The raw data supporting the conclusions of this article will be made available by the authors upon request.
